# Polytriphenylamine and Poly(styrene-*co*-hydroxystyrene) Blends as High-Performance Anticorrosion Coating for Iron

**DOI:** 10.3390/polym13101629

**Published:** 2021-05-17

**Authors:** Ting-Hsuan Lee, Jen-Hao Tsai, Hong-Yu Chen, Ping-Tsung Huang

**Affiliations:** Department of Chemistry, Fu Jen Catholic University, New Taipei City 24205, Taiwan; yes850492@gmail.com (T.-H.L.); ahahacao@gmail.com (J.-H.T.); a0930439198@gmail.com (H.-Y.C.)

**Keywords:** polytriphenylamine, poly(styrene-*co*-hydroxystyrene), blend, anticorrosion, thermal stability

## Abstract

An electroactive polytriphenylamine (PTPA-C6) is blended with poly(styrene-*co*-hydroxystyrene) (PS-*co*-PHS) as coating layers to enhance protection efficiency of PTPA-C6 on iron substrate in 3.5% sodium chloride (NaCl) solution. Experimental results show that incorporation of hydroxyl group to the polystyrene not only increases the miscibility of PTPA-C6 with PS through the hydrogen bond formation, but also enhances the bonding strength between the polymer coating layer and iron substrate. These improvements lead to superior enhancement in anticorrosion performance of PTPA-C6, even after thermal treatment. Protection efficiency (PE) of PTPA-C6 increases from 81.52% of the PTPA-C6 itself to over 94.40% under different conditions (PE_max_ = 99.19%).

## 1. Introduction

Surface functionalization of metal materials has been one of the most attractive research areas in recent years. It aims to improve the specific function of materials when interacting with their environment, especially anti-corrosion. An oxide layer is usually formed at the polymer/metal interface to protect metals from direct oxidation reactions by oxygen and moisture. However, due to the loose structure of oxide layer formed from such a direct oxidation reaction, moisture and oxygen can easily enter the inner layer of metal and react with metal continuously, leading to a severe loss of adhesion. Therefore, the metal will be seriously corroded and the polymer will be delaminated from the metal, which cause a huge impact on the economic viability of metal-based products [1,2]. In order to reduce the degree of corrosion on metals, researchers have developed various methods to control the interaction between metals and surface coating layers. Among the numerous available corrosion prevention techniques (such as electrochemical protection, corrosion inhibitor [3], and protective coating), the most common method is the application of organic coatings [4,5,6], which can provide efficient and long-lasting corrosion protection. It can generate a dense oxide passivation layer by an electrochemical reaction to limit corrosion on metal surfaces. Materials for such anti-corrosion protection can be divided into three categories-corrosion inhibitors, hydrophobic polymers, and conductive polymers. Organic inhibitors have been used as corrosion inhibitors for the protection of metallic materials against corrosion for many years. They show consistent anti-corrosion properties owing to their interaction with metals through specific functional interactions with metals. The difference of these organic compounds in inhibiting corrosion lies in their adsorption capability to the metal surface and the subsequent formation of a protective layer. In addition to the organic inhibitors, montmorillonite (MMT) [7,8,9,10] and reduced graphene oxide (RGO) [11,12,13,14,15,16] are regarded as the most representative organic additives for anti-corrosion because of their capability to lower the extent of permeation of oxygen and moisture. Montmorillonite is a commonly seen clay material used in polymer nanocomposites and RGO is a soft material composed of hydrophobic functionality. Both of them show a layered structure that can block corrosive species from the air and lower the possibility of oxidation reactions of metals. Conjugated polymers, however, exhibit an excellent anti-corrosion function against iron because they can form a thin and dense iron oxide [17,18] (FeO, Fe_2_O_3_ and Fe_3_O_4_) passivation layer on polymer/iron interface through the electrochemical reactions with iron. Heteroatom-conjugated polymers such as polypyrrole (PPy) [19,20,21], polyaniline (PANI) [22,23,24,25,26], and polythiophene (PTh) are three commonly seen model systems in corrosion protection research. Unfortunately, the mechanical strength of PANI is an issue for the long-term stability in anti-corrosion studies. The weak mechanical strength of PANI coating results in crack formation, which leads to underperformance of corrosion efficiency and poor adhesion to the substrate. Researchers incorporate inorganic nanoparticles, such as zinc oxide (ZnO) [27,28], titanium dioxide (TiO_2_) [29], and silica (SiO_2_) [30,31] into the PANI matrix to improve the mechanical properties of PANI coatings and thus enhance their long-term stability. In addition to the mechanical properties, the moisture absorption of PANI tends to lower the effectiveness of PANI as corrosion protection coating. To improve the hydrophobicity of the PANI coating, researchers have developed different approaches to resolve this issue. In terms of hydrophobicity of PANI composites, Yeh et al. [32,33,34,35] modifies PANI with MMT and RGO to limit the penetration rate of oxygen and water in PANI. These PANI/MMT and PANI/RGO blends show excellent corrosion resistibility and outstanding hydrophobicity.

Regioregular semiconducting polymer poly(3-hexylthiophene-2,5-diyl), commonly known as P3HT, has also provided good anti-corrosion properties. Hernandez–Martinez et al. Reference [36] reports the effects of thermal treatment on corrosion protection by P3HT/polystyrene (PS) and P3HT/poly(methyl methacrylate) (PMMA) blended coatings. Blends of P3HT with PS or PMMA improve protection against corrosion. However, with a thermal treatment at high temperatures, imperfections such as cracks and holes are generated in coating, which greatly reduces corrosion efficiency.

As mentioned above, conjugated polymers (such as PANI or P3HT) have been widely used in anticorrosive coatings because of their specific function to induce a thin and dense metal oxide layer on the metal surface. Furthermore, a conjugated polymer with specific functional group(s) that has good adhesion to the metal can serve as both a corrosion inhibitor and a passivation layer promoter. Based on this concept, a soluble poly triphenylamine (PTPA-C6) is synthesized and applied to the surface of iron substrate as a corrosion resistant coating by Tsai et al. [37]. Functionality such as triphenylamine contains a nitrogen atom that has been proved to be effective in corrosion protection of metals. PTPA-C6 contains triphenylamine that can undergo redox reactions easily and shows excellent anticorrosion performance on iron. However, PTPA-C6 does demonstrate good adhesion to the iron substrate. This coating still delaminates from an iron surface after being thermally-treated and the temperature exceeds 100 °C. To overcome the problem, PTPA-C6 are mixed with poly(styrene-*co*-hydroxystyrene) (PS-*co*-5PHS and PS-*co*-10PHS) to enhance the adhesion property of PTPA-C6 on iron, especially at high temperatures, and thus improve its anti-corrosion capability. With the increase of hydroxyl groups on poly(styrene-*co*-hydroxystyrene), it is expected that the nitrogen atom of PTPA-C6 can form a hydrogen bond with hydroxyl group, which improves the miscibility of the PTPA-C6/poly(styrene-*co*-hydroxystyrene) blend. The hydroxyl group can also interact with iron surfaces. Hopefully, an effective polymer blend coating on iron can not only improve the adhesion to the iron substrate, but also enhance the anti-corrosion ability of PTPA-C6 on iron, especially after thermal treatment.

## 2. Materials and Methods

### 2.1. Materials Synthesis

**Figure 1:**
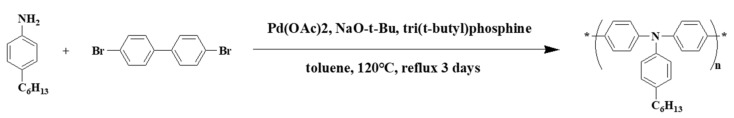
Synthetic route of PTPA-C6.

**Figure 2:**
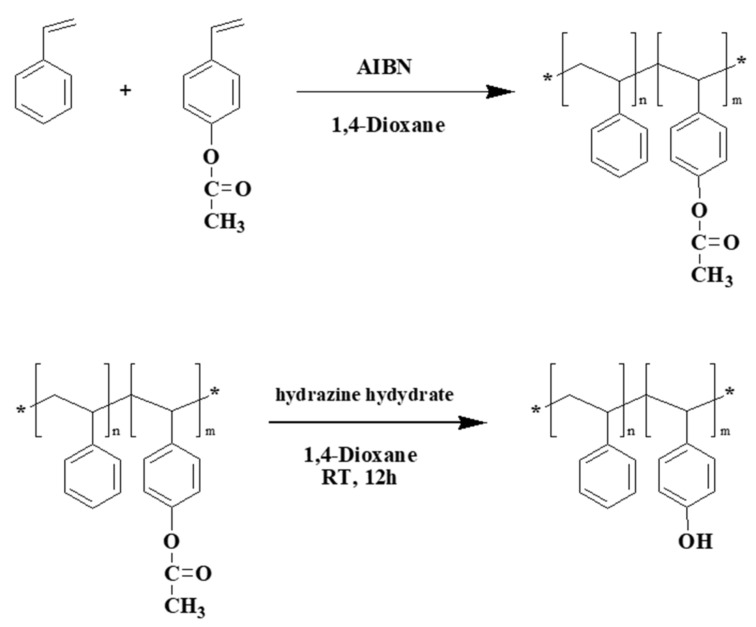
Synthetic routes of PS-*co*-PHS copolymers.

PTPA-C6 and PS-*co*-PHS were synthesized following the procedures described in the literature [37,38]. PTPA-C6 was synthesized by a reaction of 4-hexylaniline with dibromobiphenyl in anhydrous toluene (Figure 1). Meanwhile, PS-*co*-5PHS and PS-*co*-10PHS were synthesized by a reaction of an appropriate amount of styrene with acetoxystyrene in 1,4-dioxane followed by hydrolysis with hydrazine (Figure 2). ^1^H Nuclear Magnetic Resonance (NMR, 300 MHz) spectra were recorded on a Bruker AC-300 MHz (Bruker Corp., Billerica, MA, USA). Fourier-Transform Infrared spectra (FT-IR) were collected by a Perkin Elmer model 100 FT-IR spectrometer (Perkin-Elmer Co., Waltham, MA, USA). NMR spectrum and FT-IR spectrum of PTPA-C6 can be found in supporting information (Figures S1 and S2). NMR spectra of PS-*co*-5PHS and PS-*co*-10PHS are available in Figure S3. The molecular weight of polymers was measured by a Viscotek DM400/LR40 Gel Permeation Chromatography (GPC) (Malvern Panalytical, Malvern, UK) using standard polystyrene as a reference. The molecular weight of polytriphenylamine is $\bar{M_{n}}$ = 13,400/$\bar{M_{w}}$ = 27,200. The molecular weight of PS-*co*-5PHS is $\bar{M_{n}}$ = 185,000/$\bar{M_{w}}$ = 273,000. The molecular weight of PS-*co*-5PHS is $\bar{M_{n}}$ = 121,000/$\bar{M_{w}}$ = 203,000. Optical microscope photos were taken using an Olympus BH-2 Microscopy with a 40× object lens (Olympus, Tokyo, Japan) in bright field mode. Electrochemical measurements were conducted on a CHI 680C Cyclic Voltammetry (CH Instruments, Austin, TX, USA).

### 2.2. Sample Preparation

#### 2.2.1. Sample Preparation for the Optical Microscope Measurement

Glass substrates were cleaned in an ultrasonic bath using detergent, deionized water, acetone, and isopropanol sequentially. Solutions of PTPA-C6 blended with PS or PS-*co*-PHS (PS-*co*-5PHS and PS-*co*-10PHS) at a 1:1 ratio in *o*-xylene were spin-coated on glass at 500 rpm for 50 s and 1000 rpm for 100 s. They were dried at 25 and 200 °C for 1 h in air before measurement.

#### 2.2.2. Corrosion Test

Iron substrate was ground with 400, 800, and 1200 grade sandpapers and cleaned in an ultrasonic bath with hexanes after grinding. Polymer solutions in *o*-xylene were spin-coated onto iron substrates and dried at 25 °C. Thermal treatment of these samples was conducted at 100 ℃ for one hour before measurement. Thickness of the polymer coating layer was measured with an Elcometer type 456 gauge meter (Elcometer Co., Manchester, UK). The contact was is measured with a FACE contact angle meter model XP1502 (Tantec Inc., Schaumburg, IL, USA). All corrosion tests were performed in a 3.5% NaCl solution and all samples were immersed in NaCl solution for 30 min. before measurement.

## 3. Results and Discussion

### 3.1. Hydrogen Bonding Study

To study the hydrogen bond interaction between PTPA-C6 and PS-*co*-PHS copolymers, triphenylamine and *p*-cresol were used as model compounds in an NMR study.

**Figure 3:**
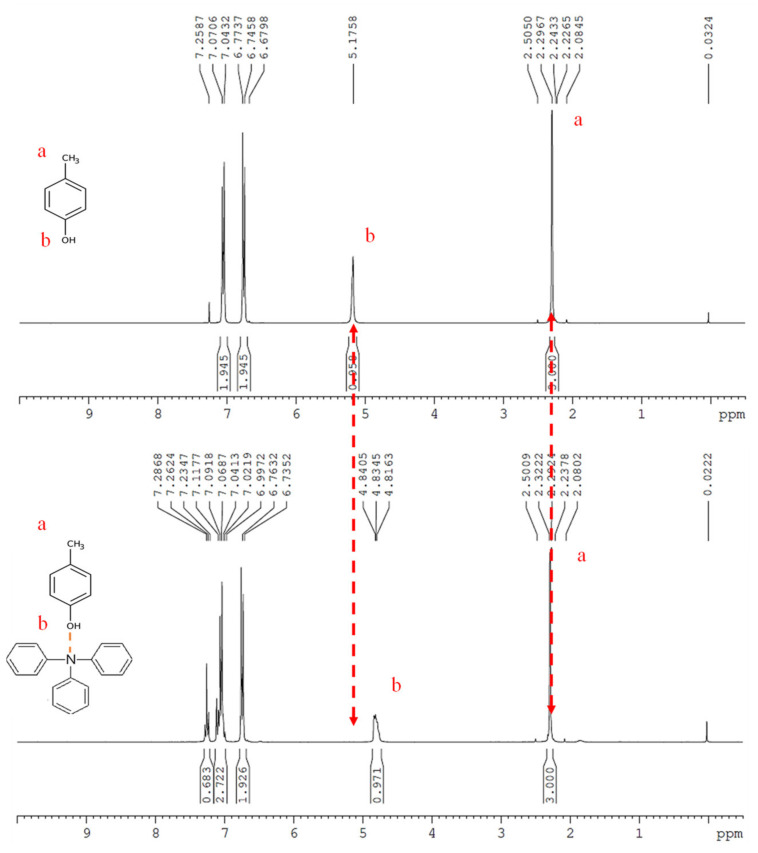
^1^H-NMR spectrum of (**a**) *p*-cresol, and (**b**) *p*-cresol/triphenylamine.

*p*-Cresol shows a characteristic chemical shift at 2.24 ppm for the methyl group and 5.18 ppm for the hydroxyl group (Figure 3a). The chemical shift of the methyl group showed a minor shift to 2.29 ppm and the hydroxyl group shifted to 4.83 ppm after mixing with triphenylamine (Figure 3b). Evidently, the hydrogen bond formation between the nitrogen atom of triphenylamine and hydroxyl group of *p*-cresol led to the spectrum shift of *p*-cresol and that enhanced the molecular interaction between triphenylamine and *p*-cresol.

Additionally, FT-IR measurements on PTPA-C6, PS-*co*-10PHS, and PTPA-C6/PS-*co*-10PHS (1:1) (Figure S4) also demonstrates the existence of H-bonding between PTPA-C6 and PS-*co*-10PHS. Free -OH peaks of PS-*co*-10PHS appear around 3500 cm^−1^ become broaden-ing after blending with PTPA-C6, which may attribute to the H-bond formation between PTPA-C6 and PS-*co*-10PHS.

### 3.2. Morphology Study

**Figure 4:**
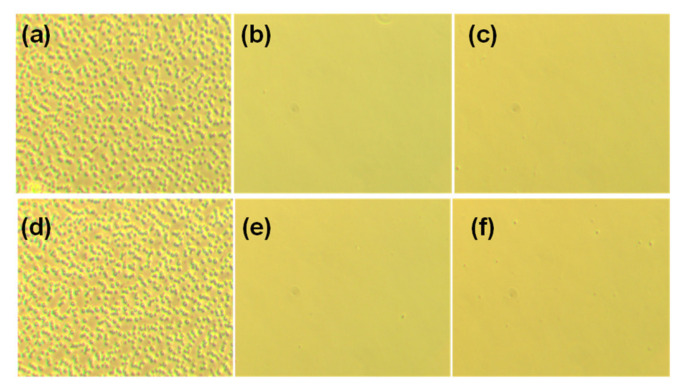
Optical microscope photos of PTPA-C6/PS blend (1:1) (**a**,**d**), PTPA-C6/PS-*co*-5PHS blend (1:1) (**b**,**e**), and PTPA-C6/PS-*co*-10PHS blend (1:1) (**c**,**f**) after spin-coating on glass and being annealed at 25 °C (**a**–**c**) and 100 °C (**d**–**f**).

Poor compatibility in polymer blends usually results in severe phase separation, especially after thermal treatment. As indicated from the NMR study, triphenyl amine shows strong H-bonding with the hydroxyl group in *p*-cresol. This result has profound effects on the compatibility of PTPA-C6/PS blend. As shown in Figure 4, the PTPA-C6/PS blend shows severe phase separation whether in the as-cast film (Figure 4a) or after thermal treatment at 100 °C (Figure 4d). With the introduction of 5 mole % hydroxyl groups to PS (PS-*co*-5PHS), compatibility between PTPA-C6 and PS improved significantly. No phase separation occurs in the PTPA-C6/PS-*co*-5PHS blend (Figure 4b), even after thermal treatment at 100 °C (Figure 4e). Similar results also appear in the PTPA-C6/PS-*co*-10PHS blend (Figure 4c,f).

### 3.3. Adhesion Test and Contact Angle Study

As mentioned earlier, corrosion protection of iron by coating with polymer can be enhanced if the penetration of moisture in polymer and adhesion of polymer to the iron substrate can be improved. PTPA-C6 exhibits good protection efficiency on iron, yet adhesion to the iron substrate needs to be further enhanced [38]. A way to lower the moisture penetration in PTPA-C6 is to increase its hydrophobicity. PS is a hydrophobic polymer and has been used to lessen the moisture uptake of PANI and P3HT. It is blended with PTPA-C6 to evaluate how PS affects the contact angle of PTPA-C6. As shown in Table 1, PTPA-C6 has a contact angle of 96.1° in the as-cast film and 98.8° after thermal treat at 100 °C. However, the contact angle of as-cast film is reduced to 93.9° after blending with PS. This can possibly be attributed to the severe phase separation, as investigated above. Delamination of the PTPA-C6/PS blend from iron substrate can also be observed in this incompatible blend. By incorporation of hydroxyl moiety to PS, the contact angle of the as-cast film increases to about 100° and it further increases to over 100° after thermal treatment. It seems that a homogeneous distribution of PS in the PTPA-C6 matrix can exhibit its hydrophobic character and increase the contact angle of PTPA-C6. In addition to the improvement in the hydrophobicity of PTPA-C6, adhesion of the PTPA-C6/PS blend is also enhanced with the incorporation of the hydroxyl group. It has been demonstrated that an iron oxides passivation layer is formed on the surface of iron substrate after coating with PTPA-C6 [38]. The interaction between PTPA-C6 and iron substrate is promoted with additional dipole-ion interaction (–OH and iron oxides), which protects the PTPA-C6/PS-*co*-PHS blends from failure in the 100 Grid test. Both PTPA-C6 and PTPA-C6+PS show clear residues in the as-cast films and annealed films in the ASTM 3359 adhesion test. The residues almost disappear in the adhesion test with the addition of a 5% or 10% hydroxyl group to the copolymer because of the enhanced interaction between the -OH group and iron oxides passivation layer.

### 3.4. Corrosion Test

**Figure 5:**
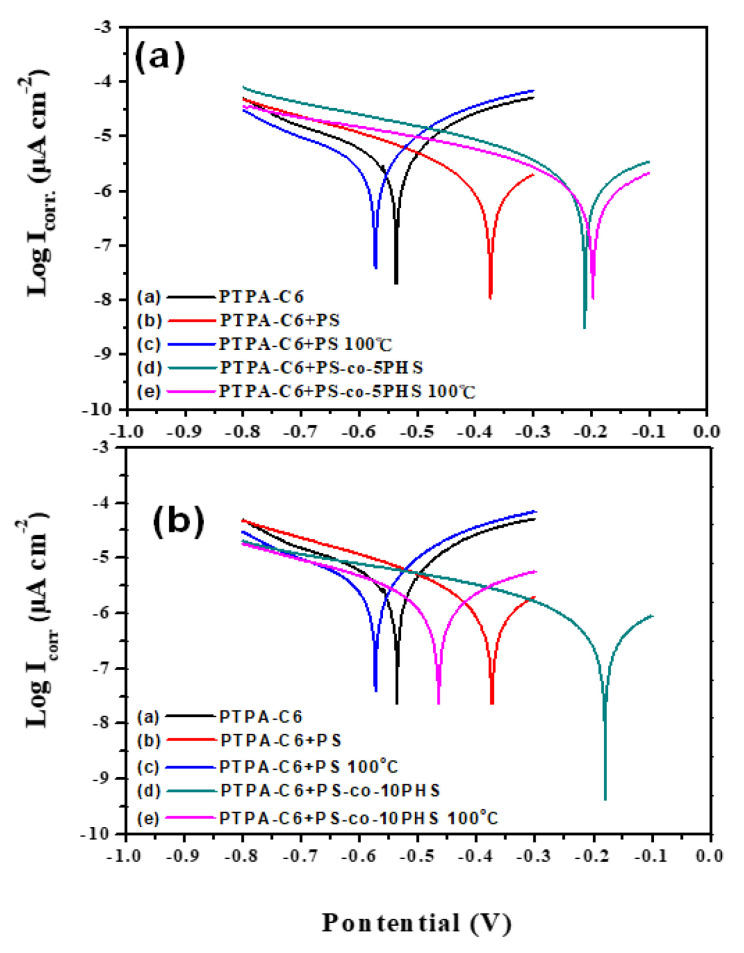
Tafel plots of (**a**) PTPA-C6 + PS-*co*-5PHS, and (**b**) PTPA-C6 + PS-*co*-10PHS at 25 °C and 100 °C.

PTPA-C6 shows good anticorrosion properties from the electrochemical impedance spectroscopy (EIS) measurements [18]. These properties are further enhanced with the increased hydrophobicity and improved bonding to the iron substrate. The corrosion current is reduced with the blending of PTPA-C6 with PS-*co*-PHS copolymers (Figure 5). As listed in Table 2, the corrosion current of PTPA-C6 on iron is 2.89 µA·cm^−2^. It increases to 3.09 µA·cm^−2^ after blending with PS and further increases to 3.60 µA·cm^−2^ after thermally treated at 100 °C because of the incompatibility between PTPA-C6 and PS, which leads to phase separation. With the introduction of hydroxyl groups to the PS, not only is compatibility between PTPA-C6 and PS improved significantly, but the bonding strength between the polymer coating layer and iron substrate is as well. Corrosion current reduces to below 1 µA·cm^−2^ in both as-cast and annealed films and also gives rise to much higher protection efficiency (PE). PE values are all above 94% after thermal annealing at 100 °C for 1 h.

**Figure 6:**
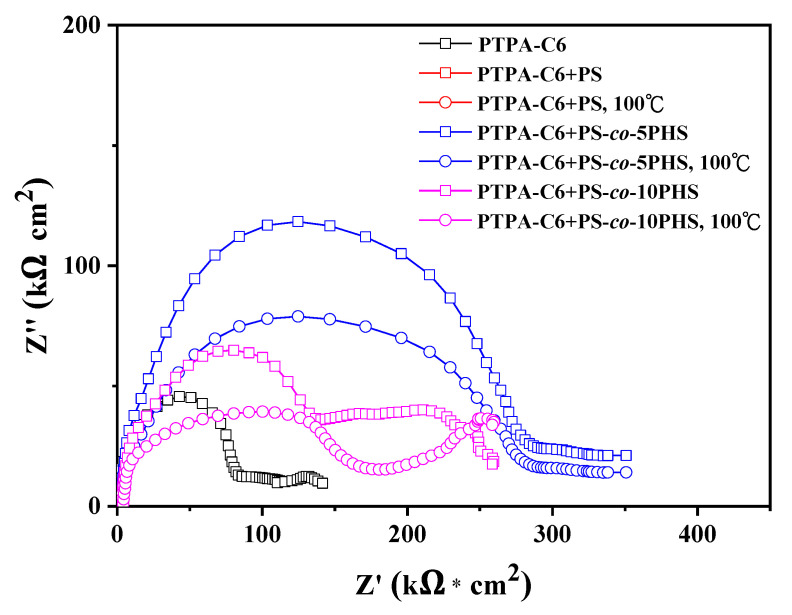
Nyquist plots of the PTPA-C6, PTPA-C6/PS, PTPA-C6 /PS-*co*-5PHS, and PTPA-C6/PS-*co*-10PHS blends coated on iron substrate in 3.5% NaCl solution.

Electrochemical impedance spectroscopy (EIS) measurement results of the above samples are shown in Figure 6. As shown from the Nyquist plot, the charge transfer resistance (R_ct_) of the polymers coated on iron substrate in 3.5% NaCl solution increases with incorporation of the hydroxyl group to the PS. Although the R_ct_ shows some variation depending on the extent of miscibility between the PTPA-C6 and PS-*co*-PHS copolymers, the PTPA-C6/PS-*co*-PHS blends perform much better than that of the PTPA-C6 and PTPA-C6/PS blend. However, the R_ct_ tends to decrease upon thermal annealing. It is possible that thermal treatment at high temperature (100 °C) lowers the strength of hydrogen bonding and decreases the extent of miscibility between PTPA-C6 and PS-*co*-PHS blends.

## 4. Conclusions

With the introduction of hydroxyl group to the polystyrene, not only the does intermolecular bonding strength between PTPA-C6 and PS-*co*-PHS copolymers increase, but the bonding of polymer coating layer to the iron substrate does as well. This improvement leads to enhanced anticorrosion performance of the PTPA-C6/PS-*co*-PHS blends, as revealed from the corrosion test and EIS measurement.

## Figures and Tables

**Table 1:** Adhesion tests (ASTM 3359) and contact angle measurements of water on PTPA-C6 and its blends at room temperature (25 °C) and under a 100 °C treatment.

Coating Layer	100 Grid Test	Contact Angle (°)
PTPA-C6, 25 °C	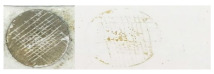	96.1 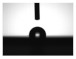
PTPA-C6, 100 °C	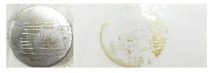	98.8 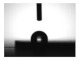
PTPA-C6 + PS, 25 °C	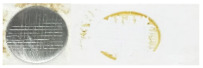	93.9 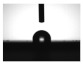
PTPA-C6 + PS, 100 °C	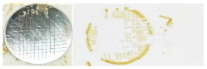	99.2 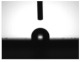
PTPA-C6 + PS-*co*-5PHS, 25 °C	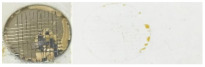	99.5 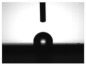
PTPA-C6 + PS-*co*-5PHS, 100 °C	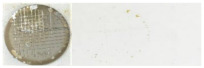	102.3 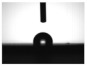
PTPA-C6 + PS-*co*-10PHS, 25 °C	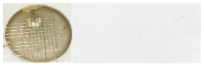	99.7 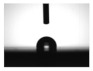
PTPA-C6 + PS-*co*-10PHS, 100 °C	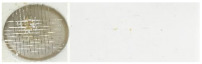	101.4 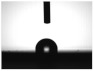

**Table 2:** Electrochemical properties of PTPA-C6, PTPA-C6/PS, PTPA-C6/PS-*co*-5PHS, and PTPA-C6/PS-*co*-10PHS blends coated on iron substrates for EIS measurement in 3.5% NaCl solution.

Coating Layer ^a^	R_p_ (kΩ cm^2^)	I_corr_ (μA/cm^2^)	R_corr_ (MPY ^c^)	PE ^b^ (%)	Thickness (μm)
Bare-Fe	4.20	15.38	7.02	-	-
PTPA-C6, 25 °C	160.25	2.84	1.30	81.52	14
PTPA-C6 + PS, 25 °C	32.80	3.09	1.41	79.88	12.8
PTPA-C6 + PS, 100 °C	10.57	3.60	1.64	76.67	13.5
PTPA-C6 + PS-*co*-5PHS, 25 °C	417.00	0.15	0.07	98.97	18
PTPA-C6 + PS-*co*-5PHS, 100 °C	372.41	0.86	0.39	94.40	10.7
PTPA-C6 + PS-*co*-10PHS, 25 °C	284.93	0.12	0.06	99.19	12.7
PTPA-C6 + PS-*co*-10PHS, 100 °C	246.04	0.30	0.14	97.99	12.6

^a^: blend ratio = 1/1, ^b^: corrosion protection efficiency, ^c^: mils per year (corrosion rate).

## Data Availability

Data are available through the corresponding author.

## Supplementary Materials

The following are available online at https://www.mdpi.com/article/10.3390/polym13101629/s1, Figure S1: NMR spectrum of PTPA-C6, Figure S2: FT-IR spectrum of PTPA-C6, Figure S3: NMR spectra of (a) PS/PS-*co*-5PAS/PS-*co*-5PHS; (b) PS/PS-*co*-10PAS/PS-*co*-10PHS, Figure S4: FT-IR spectra of PTPA-C6, PS-*co*-10PHS, and PTPA-C6/PS-*co*-10PHS (1:1).
