# Supplementary material for: Polytriphenylamine and Poly(styrene-co-hydroxystyrene) Blends as High-Performance Anticorrosion Coating for Iron
Source: Polymers (Basel). 2021 May 17;13(10):1629. doi: 10.3390/polym13101629 (PMC8156867; doi:10.3390/polym13101629)
Supplement: Supplementary file 1 [file polymers-13-01629-s001.zip › polymers-1177781-supplementary.pdf]

Supporting Information

Polytriphenylamine and Poly(styrene-co-hydroxystyrene) Blends as High-performance Anticorrosion Coating for Iron

Ting-Hsuan Lee,[1] Jen-Hao Tsai,[1] Hong-Yu Chen,[1] and Ping-Tsung Huang[1,*]

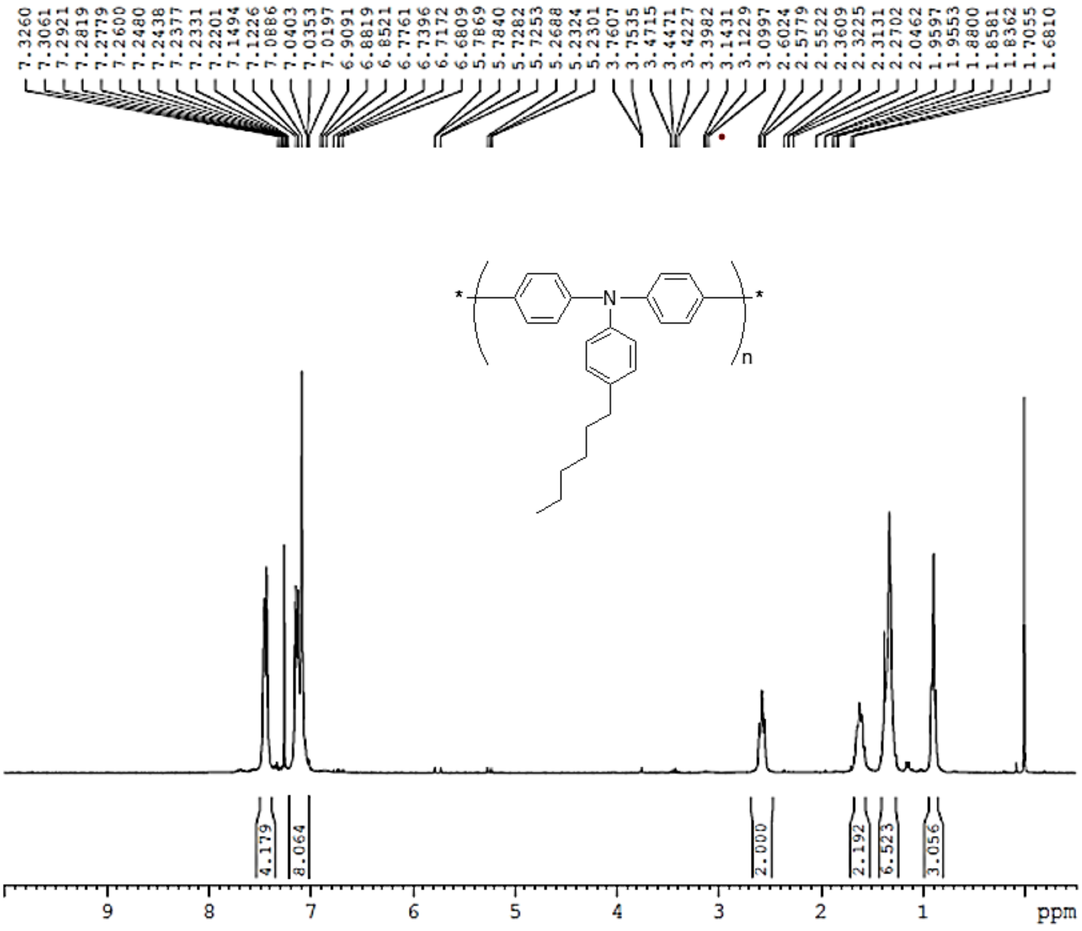

Figure S1. NMR spectrum of PTPA-C6

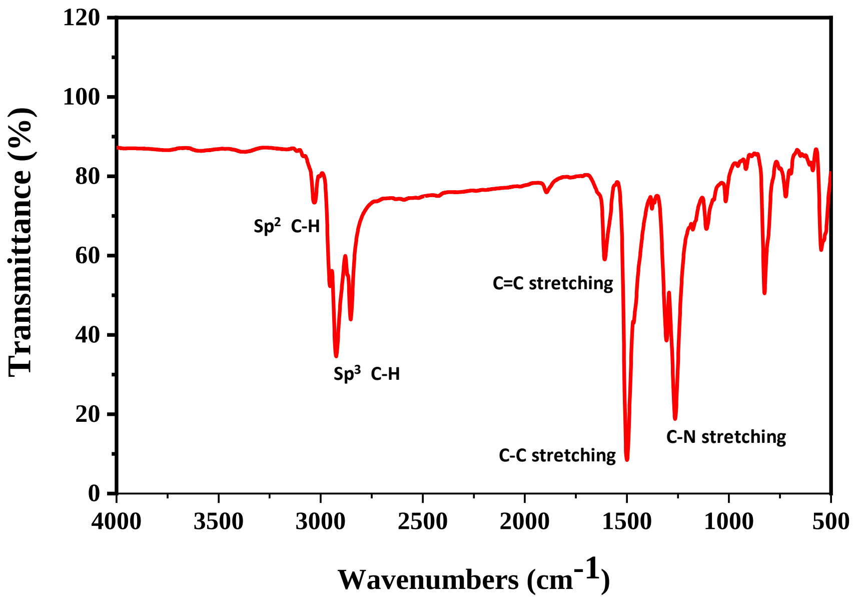

Figure S2. FT-IR spectrum of PTPA-C6

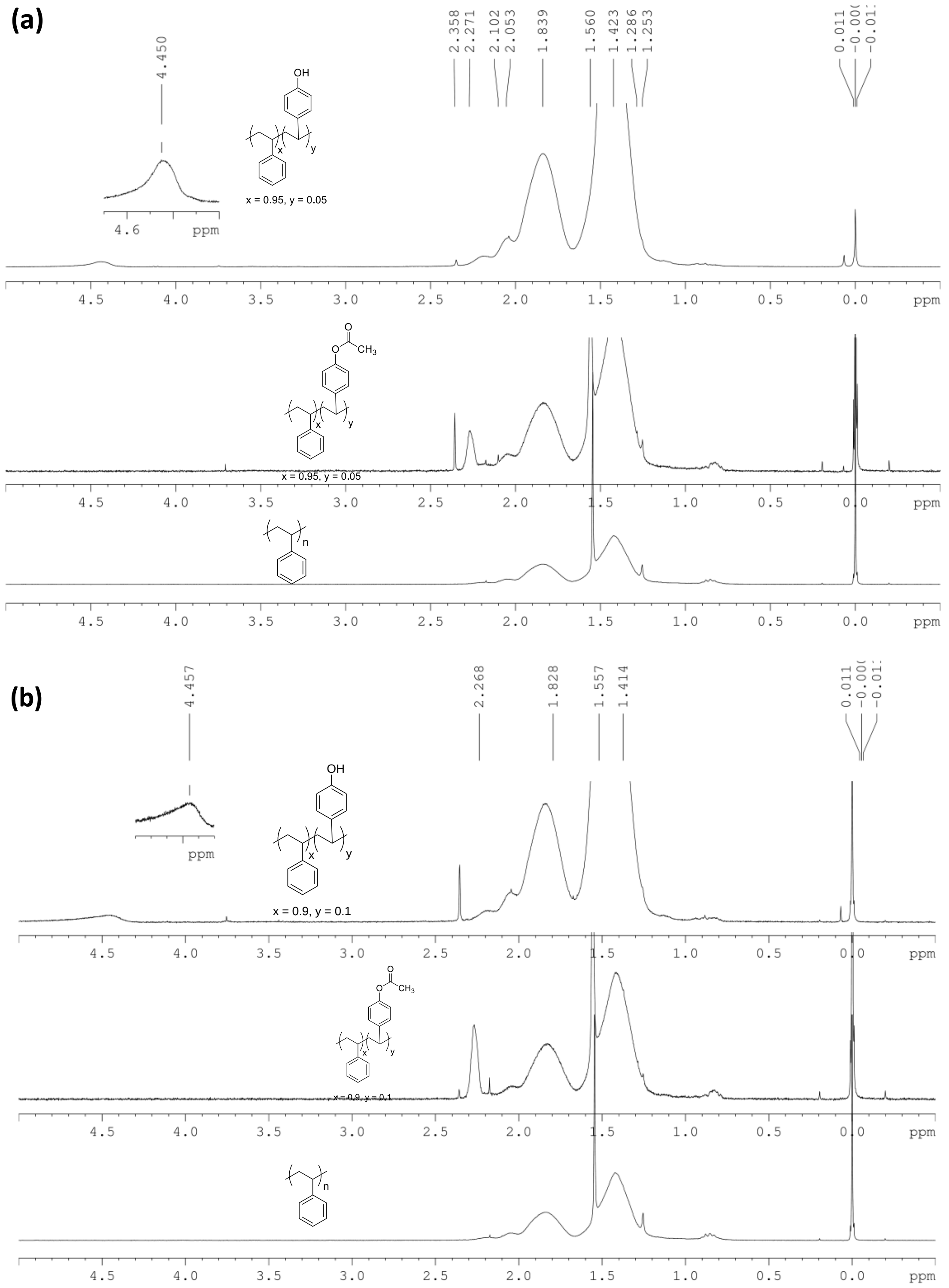

Figure S3. NMR spectra of (a) PS/PS-co-5PAS/PS-co-5PHS ; (b) PS/PS-co-10PAS/PS-co-10PHS

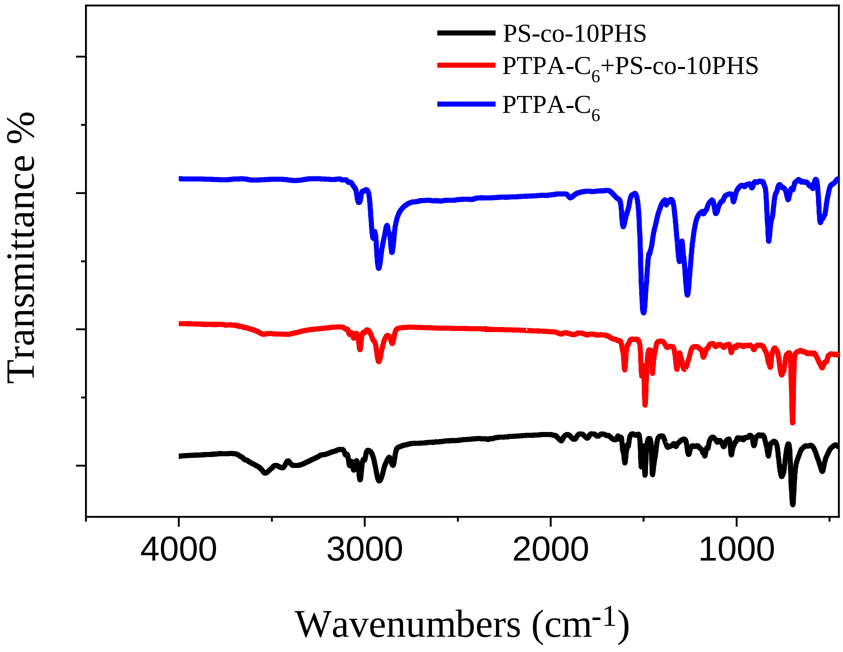

Figure S4. FT-IR spectra of PTPA-C6, PS-co-10PHS, and PTPA-C6/PS-co-10PHS (1:1)
